# Pediatric immune-mediated necrotizing myopathy

**DOI:** 10.3389/fneur.2023.1123380

**Published:** 2023-03-20

**Authors:** Chen-Hua Wang, Wen-Chen Liang

**Affiliations:** ^1^Department of Pediatrics, Kaohsiung Municipal Hsiao-Kang Hospital, Kaohsiung, Taiwan; ^2^Department of Pediatrics, Kaohsiung Medical University Hospital, Kaohsiung Medical University, Kaohsiung, Taiwan; ^3^Department of Pediatrics, School of Medicine, Kaohsiung Medical University, Kaohsiung, Taiwan; ^4^Graduate Institute of Clinical Medicine, College of Medicine, Kaohsiung Medical University, Kaohsiung, Taiwan

**Keywords:** immune-mediated necrotizing myopathy, HMGCR, SRP, idiopathic inflammatory myopathy, juvenile myositis

## Abstract

Immune-mediated necrotizing myopathy (IMNM) is a type of inflammatory myopathy. Most patients with IMNM produce anti-3-hydroxy-3-methylglutaryl coenzyme A reductase or anti-signal-recognition particle autoantibodies. IMNM is much rarer in children than in adults. We conducted this mini review focusing on pediatric IMNM to present current evidence regarding its epidemiology, clinical characteristics, diagnosis, and treatment. Our findings indicate that pediatric IMNM often causes severe muscle weakness and is refractory to corticosteroids alone. Furthermore, delayed diagnosis is common because of the clinicopathological similarity between IMNM and inherited myopathy. Raising awareness regarding pediatric IMNM may facilitate early diagnosis and effective treatment.

## 1. Introduction

Idiopathic inflammatory myopathy (IIM) is a group of autoimmune disease causing muscle inflammation with/without other organ involvement. Initially, IIM is categorized into dermatomyositis (DM) and polymyositis (PM) only (1). The patients with IIM presented with limb girdle weakness, often with myalgia and sometimes with dysphagia or respiratory involvement. Disease course typically progresses in weeks or months. The way to differentiate DM from PM is only by the presence of characteristic skin rash and perifascicular atrophy on muscle pathology. In 1995, IBM was established by clinical course with relatively slow onset and characteristic pathological features including inflammation, vacuoles, amyloid deposits, and 15–18 nm tubulofilament inclusions (2). In the past decade, anti-sythetase syndrome (ASS) and immune-mediated necrotizing myopathy (IMNM) have been determined as new subgroups of IIM by clinical, pathological and serological characters. After the establishment of new disease category, PM seems to become a diagnosis of exclusion and continuous use of this disease name may need further debate (3).

IMNM is characterized by acute onset of proximal muscle weakness, markedly elevated creatine kinase levels, and muscle pathology delineated by necrotic, regenerating fibers and scarce lymphocyte infiltration. On the basis of the seroantibodies involved, IMNM can be divided into the following three subtypes: anti-3-hydroxy-3-methylglutaryl coenzyme A reductase (HMGCR) myopathy, anti-signal recognition particle (SRP) myopathy, and seronegative myopathy. In 2010, anti-HMGCR antibody was first identified as autoantibody in patients with necrotizing myopathy. Notably, most antibody-positive patients had a history of statin exposure (4). Thus, anti-HMGCR myopathy was initially considered to be associated with statin exposure and to mainly affect adults based on the hypothesis of upregulated expression of HMGCR by statin (5). However, as more cases of anti-HMGCR myopathy were reported in statin-naive children, the pathomechanism was speculated to be different in the pediatric population. In general, pediatric patients with IMNM exhibit more insidious onset, treatment resistance, and unfavorable outcomes than adults. Due to a relatively slow onset and a dystrophic pattern of muscle pathology, IMNM is often misdiagnosed as muscular dystrophy (6); the diagnosis of IMNM is therefore challenging to make in pediatric patients. With regard to treatment, corticosteroids alone may exhibit low efficiency in the treatment of pediatric IMNM. Most pediatric patients require combination therapy including intravenous immunoglobulin (IVIg) and immunosuppressants, such as rituximab, cyclophosphamide, and methotrexate (MTX). Early diagnosis and prompt treatment are necessary for better outcomes and minimal permanent disability. In this review, we would present current understanding and evidence s of the epidemiology, clinical manifestations, diagnosis, and treatment, focusing on pediatric IMNM.

## 2. Epidemiology

In juvenile IIM, the most common disease is juvenile dermatomyositis which accounts for 75% (7) of patients. Pediatric IMNM is a relatively rare subgroup. In one study with 440 juvenile IIM patients, pediatric IMNM accounts for only 2.9% in juvenile IIM (8). Remarkably, a relatively high IMNM proportion (21%) was reported by an Asian juvenile IIM study (9). Among pediatric IMNM, the proportions of Anti-HMGCR myopathy and anti-SRP myopathy are reported 38.4 and 61.5%, respectively (8). The proportion of seronegative subgroup in pediatric IMNM is difficult to estimate as only two case reports have been published so far (10, 11).

IMNM seems to make up larger proportion of adult IIM, which accounts for 10–38% (12, 13). Previous studies including mainly adult IMNM patients showed that proportion of anti-HMGCR myopathy, anti-SRP myopathy and seronegative IMNM were 21.8–54%, 28.5–44%, and 13–40%, respectively (13–18). The difference of prevalence from pediatric IMNM patients might come from the underdiagnosis of pediatric IMNM and longer investigation time is necessary.

Statin exposure was previously hypothesized to be a trigger for anti-HMGCR myopathy; studies conducted in adult patients reported that 15–65% of adult patients were exposed to statins (5, 19). However, our literature review revealed no reports of statin use in pediatric patients with anti-HMGCR myopathy. In addition to medications, statin is present in mushrooms (20), red yeast rice (21), and Pu-erh tea (22), which are commonly used in Asian dishes. A study reported the exacerbation of adult anti-HMGCR myopathy after mushroom intake (23). Therefore, food-derived statin may be a risk factor for anti-HMGCR myopathy in pediatric patients who usually would not receive statin-based medications.

## 3. Human leukocyte antigen typing

Human leukocyte antigen (HLA) typing is strongly correlated with several autoimmune diseases; for example, HLA DRB1*11:01 is a risk factor for adult anti-HMGCR myopathy (24–26). By contrast, a study on juvenile anti-HMGCR myopathy revealed that all pediatric patients carried DRB1*07:01 but none carried DRB1*11:01 (8). In another study, DRB1*08:03 was frequently detected in Japanese patients with anti-SRP myopathy compared with the findings in the control group (25). The prevalence of DRB1*08:03 is relatively high in the Japanese population (27). In a Korean study, DRB1*14:03 was associated with anti-SRP myopathy (28). Thus, HLA typing may explain the geographical difference in the prevalence of IMNM.

## 4. Clinical manifestation

The most common clinical symptoms of IMNM is progressive proximal muscle weakness, especially lower extremities (3). Weakness often started from lower extremities, whereas initial presentation with upper extremities weakness was ever reported in about one-fifth of patients by one Japanese study (29). The severity of muscle involvement is often more than other IIM (3, 5, 16). Marked elevated creatine kinase (often ranging from 4,000 to 8,000 IU/L (30)) is a characteristic finding, and it is also a biomarker of disease activity (31). Only one-third of IMNM patients have insidious-onset (> 6 months) disease course. Most of IMNM patients are with onset less than 6 months (17).

Because of its relatively low prevalence, large-scale case series on pediatric IMNM are rare. We reviewed the literature and summarized the clinical manifestations of pediatric patients (Table 1), excluding those for whom detailed data were unavailable. A total of 84 pediatric patients with IMNM were included; of them, 43 had anti-HMGCR myopathy and 41 had anti-SRP myopathy. Female predominance (female/male = 3.2:1) was noted, and the ratio was slightly higher than that noted in adult patients (1.69–2.75:1) (13, 17). The average onset age was 9.4 years (*n* = 49), and the youngest patient was 10 months old (9). The most common clinical pattern in pediatric patients is a subacute onset [1–3 months: 41% (28/68)] followed by an insidious course [>6 months: 35% (24/68)]. Because the disease onset and course could be slow, a timely and accurate diagnosis is difficult. In a case series (17), approximately 50% of pediatric IMNM patients were misdiagnosed with limb girdle muscular dystrophy (LGMD). Another study revealed that 55% of all patients (6/11, including children and adult patients) had a clinical diagnosis of LGMD but tested negative on genetic examination; notably, anti-HMGCR antibody were detected in their blood samples (6). Other than LGMD, IMNM might also resemble facioscapulohumeral muscular dystrophy due to a winged scapula and shoulder girdle involvement (41, 42). Therefore, autoantibody assessment is recommended for patients with suspicious muscular dystrophy without any positive genetic test results or family history.

**Table 1:** Clinical characteristics of pediatric IMNM.

Reference	Number of patients	Autoantibody	Onset age*	Gender	Onset pattern	dysphagia	Lung disease	Heart involvement	Skin lesion	Initial CK	Initial diagnosis	Treatment
Suarez et al. (32)	1	HMGCR	4 Years	F	Slow	-	-	-	-	7,842	MD	IVIg
Liang et al. (9)	9	HMGCR	10 M ~ 13 Years	4M; 5F	Insidious: 5	NA	0/9	0/9	22%	918–10,891	4IM;3 MD	IVIg/CS/Tacrolimus/MTX/MMF
					Subacute: 4				(2/9)		1CMD;1DM	
Tansley et al. (33)	4	HMGCR	4 ~ 13 Years	1M; 3F	Insidious: 1	25%	NA	NA	50%	12,000–44,000	1DM	MTX/CS/ IFX; IVMP/CYP/MTX/AZA/RTX/IFX
					Subacute: 1	(1/4)			(2/4)		1IM	MTX/RTX
					Slow: 2							IVMP/CS/MTX/IFX/IVIg/MMF/CYP
Mohassel et al. (6, 34)	2	HMGCR	8–10 Years	1 M; 1F	Insidious: 2	NA	50%	NA	50%	8,000–23,000	1 MD	IVIg
							(1/2)		(1/2)			
Velardo et al. (35)	1	HMGCR	7 Years	M	Subacute: 1	+	+	+	+	10,000	IMNM	IVMP/IVIg/MTX
Kishi et al. (8)	5	HMGCR	Diagnosis: 7.1–12 Years	3F	Subacute: 2	60%	40%	20%	80%	NA	IMNM	MTX/IVMP/IVIg/CsA/AZA/MMF/CYP/RTX/abatacept/anti TNF
				2M	Slow: 3	(3/5)	(2/5)	(1/5)	(4/5)			
	8	SRP	Diagnosis: 10.7–16 Years	5F	Insidious: 2	50%	75%	50%	>50%	NA	IMNM	NA
				3M	Slow: 1	(4/8)	(6/8)	(4/8)	(>4/8)			
					Subacute: 4							
Della Marina et al. (36)	1	HMGCR	9 Years	F	Subacute	NA	NA	−	+	18,000	DM	IVMP/MTX/CS/ETN/IVIg/MMF/ CYP/RTX
	1	SRP	8 Years	F	Insidious	+	+	−	+	10,710	MD	IVMP/MTX/IVIg/RTX
Tard et al. (37)	1	HMGCR	5 Years	F	NA	+	+	−	+	7,576	CMD	IVIg
Allenbach et al. (17)	8	HMGCR	4–16 Years	8F	Insidious: 4	NA	NA	NA	NA	NA	4 IM; 4LGMD	NA
					NA:4							
Jiao et al. (38)	4	HMGCR	6–14 Years	3F	Insidious: 2	0/4	NA	NA	50%	8,687–9,786	IMNM	CS/IVIg/AZA/ tripterygium wilfordii
				1M	Slow: 2				(2/4)			HC/MTX/LFM/CYP/MMF/CsA/RTX
												CS/IVIg/CsA/CYP/RTX/MTX
Tanboon et al. (39)	1	HMGCR	5 Years	F	Insidious: 1	NA	NA	LVH	NA	8,452	Myofibrillar myopathy	CS/IVIg/MTX
Mekmangkonthong et al. (40)	1	HMGCR	9 Years	M	Acute: 1	−	−	−	−	30,833	IMNM	CS/IVMP/IVIg/MTX
Suzuki et al. (41)	2	SRP	5–9 Years	2F	Insidious: 2	NA	NA	NA	NA	2,410–2,467	1FSHD	CS
												CS/MTX/CYP/Tacrolimus
Suzuki et al. (42)	1	SRP	11 Years	M	NA	+	−	NA	−	4,180	FSHD	CS
Kawabata et al. (43)	1	SRP	15 Years	F	subacute:1	+	NA	NA	−	6,970	IMNM	CS/CYP/AZA/PE
Zhao et al. (44)	3	SRP	4–11 Years	3F	Subacute: 1	33%	0/3	0/3	NA	4,020–13,265	IMNM	IVIg/CS
					Slow: 1	(1/3)						
					Insidious: 1							
Luca et al. (45)	1	SRP	12 Years	F	Subacute: 1	+	−	NA	+	8,826	IMNM	IVMP/AZA/MTX/RTX/LFM/IVIg
Momomura et al. (46)	1	SRP	15 Years	F	Subacute: 1	+	NA	NA	−	20,375	IMNM	IVMP/PE/CYP/CS/AZA
Kobayashi et al. (47)	1	SRP	6 Years	M	Insidious: 1	−	−	−	−	5,896	LGMD	IVMP/CS/MTX/IVIg/Tacrolimus
Suzuki et al. (29)	8	SRP	NA	NA	NA	NA	−	NA	NA	NA	NA	NA
Binns et al. (48)	3	SRP	11–14 Years	3F	Acute: 1	33%	100%	0/3	33%	19,808–25,937	IMNM	IVMP/CS/MTX/RTX/CYP/IVIg
					Subacute: 1	(1/3)	(3/3)		(1/3)			
					Slow: 1							
Rider et al. (49)	1	SRP	10 Years	F	Slow: 1	NA	+	−	+	8,316	IMNM	CS/MTX/IVIg
Rider et al. (50)	6	SRP	11.6–16.1 Years	4F	Insidious: 2	50%	83%	50%	66%	9,111 ~ 22,857	IMNM	NA
				2 M	Slow: 1	(3/6)	(5/6)	(3/6)	(>4/6)			
					Subacute: 3							
Rouster-Stevens et al. (51)	3	SRP	11–16 Years	3F	Subacute: 2	33%	66%	66% (2/3)	66%	22,155–33,000	IMNM	IVMP/CS/HC/CYP/tacrolimus/MMF/IFX
					Slow: 1	(1/3)	(2/3)		(2/3)			IVIg/CS/MTX/MMF/CYP/IFX/IVMP/CYP
												CS/MTX/IFX/CsA/AZA
Takada et al. (52)	1	SRP	17 Years	F	NA	NA	−	NA	+	6,453	DM	NA
Hou et al. (53)	5	HMGCR	4–15 Years	5F	Subacute: 5	20%	0/5	0/5	80%	4,231–38,966	IMNM	1CS
						(1/5)			(4/5)			3CS/IVIg/MTX
												1CS/IVIg/MTX/AZA/CYP
Total	84	43HMGCR	Average:	58F	Acute: 2	25%	27%	14%	39%		15 patients were diagnosed as CMD/MD initially	
		41SRP	9.4Y (*n* = 49※)	18 M	Subacute: 28	(21/84)	(23/84)	(12/84)	(33/84)			
				8NA	Slow: 14							
					Insidious: 24							
					NA: 16							

IM, inflammatory myopathy; MD, muscular dystrophy; CMD, congenital muscular dystrophy; DM, dermatomyositis; LGMD, limb girdle muscular dystrophy; FSHD, facioscapulohumeral muscular dystrophy; NA, not available. CS, Corticosteroid; IVMP, intravenous methylprednisolone; MTX, methotrexate; CYP, cyclophosphamide; RTX, rituximab; MMF, mycophenolate mofetil; HC, hydroxychloroquine; CsA, cyclosporine; ETN, etanercept; IFX, infliximab; AZA, azathioprine; LFM, leflunomide; PE, Plasma exchange.

* Onset pattern: acute:<1 month; subacute 1–3 months; chronic 3–6 months; insidious >6 months.

※ Exclude Kishi (8), Allenbach (17), Suzuki (29), and Rider (50) due to lack of detailed data.

Compared to anti-HMGCR myopathy, anti-SRP myopathy is accompanied by more frequent neck weakness, bulbar dysfunction (e.g., dysphagia), cardiac involvement, and interstitial lung disease (8, 13, 29, 36, 43). Cardiac abnormality has been reported in only three pediatric patients with anti-HMGCR myopathy, including hypokinesia, rigidity over interventricular septum basal segments, and left ventricular hypertrophy (8, 35, 39).

Cancer risk is much higher in cases of seronegative IMNM than anti-SRP myopathy. The association between cancer and anti-HMGCR myopathy is debatable, although some studies have reported a positive relationship (54, 55). However, to the best of our knowledge, no pediatric patient with IMNM has been diagnosed with concurrent malignancy.

Cutaneous involvement is not uncommon in IMNM. Per existing case reports, it is most common among pediatric patients. However, a recent study indicated that 56% of all adult patients with anti-HMGCR have skin lesions (56). Our review revealed that 39% (33/84) of all pediatric patients with IMNM exhibited cutaneous manifestations (Table 1). The manifestations include rash, urticaria, alopecia, cracked fingertip, palm desquamation, mechanic’s hands, linear scleroderma morphea, linear extensor erythema, periungual exanthema, periungual capillary abnormality, malar rash, shawl rash, Gottron’s papules, heliotrope rash, Raynaud phenomenon, hypopigmentation, and interstitial granulomatous dermatitis (8, 9, 33–38, 45, 48, 49, 51–53).

In some patients, IMNM developed or worsened after an infection (36, 43, 46, 48, 51, 57). The most common event preceding IMNM development was respiratory tract infection; in addition, lymphadenitis (43) and dengue fever (40) were ever reported to be preceding events in pediatric patients. The interval between an infection and muscle weakness onset is 1–4 weeks. A case report published during the COVID-19 pandemic reported that an adult patient with anti-HMGCR myopathy experienced disease relapse after SARS-CoV-2 infection (58). Another adult patient developed anti-SRP myopathy after receiving the Pfizer–BioNTech vaccine (59). The association between infection and IMNM remains unknown; therefore, further studies are necessary to elucidate the pathomechanism of IMNM.

Pediatric seronegative IMNM is very rare and there were only two reported cases (10, 11). Their onset age were 10 years old and 11 years old, respectively. Both of them showed bilateral proximal symmetric weakness over extremities. One patient developed myopericarditis and cardiogenic shock after mild clinical improvement toward initial treatment including steroid pulse therapy/IVIg/MTX. Although the case number is too small to compare with seropositive pediatric IMNM patients, it seems that cardiac evaluation is essential in these patients.

## 5. Diagnosis

### 5.1. Imaging

Although muscle imaging is not included in IMNM diagnostic criteria, it has emerged to be an important tool for differential diagnosis among IIMs. Recent accumulation of evidence demonstrated that muscle magnetic resonance imaging (MRI) is helpful for IMNM evaluation. The muscle MRI for IMNM typically reveals prominent muscle edema, atrophy, and fatty infiltration, primarily over the thigh muscle (60, 61). The most severely fatty infiltrated region are hamstrings and adductor magnus while quadriceps femoris is the least (61). Pelvic and adductor involvement is common and usually severe during the early period of the disease course. By contrast, these regions are often unaffected in patients with dermatomyositis (62). In addition, adductor brevis edema and obturator externus atrophy are unique common findings in IMNM (60). Shoulder involvement with a clinical scapular winging has been observed in some patients with IMNM (6, 63); however, the frequency of this phenomenon is relatively low in other IIMs. Fascial edema in the semitendinosus was very rare in IMNM, in contrary to DM (60). Compared with patients with anti-HMCGR myopathy, those with anti-SRP myopathy exhibit marked atrophy and fatty infiltration on muscle imaging (60), which are consistent with their clinical symptoms. The distribution of muscle involvement seems to be no different between pediatric and adult IMNM patients.

### 5.2. Muscle pathology

Myo-pathological analysis plays an important role in differential diagnosis of IIM. Sarcoplasmic major histocompatibility complex class I (MHC-I) staining is usually observed in all IIM but the combination use of some other immunohistochemical staining might make the subtypes of IIM distinguishable. In DM, in addition to classical perifascicular atrophy, sarcoplasmic myxovirus resistance protein A (MxA) expression especially in perifascicular areas has been recently identified as a pathological hallmark. Membrane attack complex (MAC; C5b-9 complement complex) is often deposited on endomysial capillaries, not sarcolemma of myofibers. MHC-II is negative. In ASS, perifascicular necrosis with perimyisial connective tissue fragmentation is often observed which might look like perifascicular atrophy, but contrary to DM, MxA is negative and MAC deposition could be present on sarcolemma; MHC-II expression is characteristically observed on the sarcolemma of many myofibers. In IBM, there are fibers with p62-positive coarse aggregates in the sarcoplasm. Endomysial infiltrating lymphocytes are positive for CD8. MHC-II staining on both the sarcolemma and the sarcoplasm of myofibers is present (64, 65).

The typical muscle pathologies associated with IMNM are as follows: necrosis and regeneration of muscle fibers; elevated expression levels of MHC-I, but not MHC-II, on the sarcolemma; frequent deposition of MAC on the sarcolemma (14, 66); infiltration of lymphocytes to the endomysium but rare invasion of healthy myofibers; and fine granular patterns of p62 (autophagy marker) over the sarcolemma (14, 66–69). In some pediatric patients with a prolonged disease duration, increased endomysial fibrosis, increased fiber size variability, and internal nuclei have been observed (34, 35, 37). Because of the pathological similarity between IMNM and muscular dystrophy, initial misdiagnosis is common. Thus, immunohistochemical staining is crucial for IMNM diagnosis. Because MHC-I expression indicates muscle damage, it is a sensitive, but not a specific marker, for IMNM (70). The deposition of MAC on the sarcolemma and a fine granular pattern of p62, a positive autophagy marker in sarcoplasm are regarded as characteristic features but lack of specificity (67, 71).

## 6. Treatment

In 2022 British Society for Rheumatology guideline for juvenile IIM (72), it recommended that combination of high dose corticosteroids and methotrexate should be used as first-line treatment in most pediatric cases. Although corticosteroid is essential, it should be weaned when disease activity improved (often 6 weeks after treatment initiation). For pediatric with myositis and skin lesion, mycophenolate mofetil is an alternative treatment option. Intravenous immunoglobulin, rituximab or cyclophosphamide may be considered in severe and/or refractory IIM. Clinician should treat juvenile onset IIM aggressively to achieve early, complete recovery of muscle weakness and inflammation to improve outcome.

Among juvenile IIM, juvenile DM have favorable treatment response. Previous literature showed that the probability of corticosteroid discontinuation was 56%, complete clinical response 38% and remission 30% by 5 years after initial treatment for juvenile DM (73). However, pediatric IMNM is often refractory to treatment. Because of the low prevalence of pediatric IMNM, few randomized controlled trials have focused on its treatment options. In adult patients, treatment is prescribed primarily on the basis of the attending physician’s experience or expert consensus. Corticosteroids are often used as the initial therapeutic agent for pediatric IMNM. Initially, prednisolone is administered at a dose of 1 or 2 mg/kg/day; then, intravenous pulse therapy may be added to the treatment regimen considering disease severity. However, corticosteroids alone may not be an effective treatment option because most pediatric patients require additional immunosuppressants for disease management. In a study (8) comprising five pediatric patients with anti-HMGCR myopathy, the patients required the simultaneous administration of an average of 2.4 therapeutic agents and 8 different medication trials during the entire disease course.

Also by the consensus of the European Neuromuscular Center (ENMC) (14) meeting, the initial treatment for IMNM should include corticosteroids combined with additional agents (simultaneous administration or within 1 month). In general, MTX is the first choice of treatment. Rituximab and IVIg may be combined with MTX or used instead of MTX for patients with anti-SRP myopathy and patients with anti-HMGCR myopathy, respectively.

Other immunosuppressants used in some pediatric patients include cyclophosphamide, azathioprine, mycophenolate mofetil, infliximab, tacrolimus, cyclosporine, leflunomide, etanercept, abatacept, anti-tumor necrosis factor, and hydroxychloroquine. In two female pediatric patients with anti-SRP myopathy, plasma exchange was performed in addition to the simultaneous administration of immunosuppressants. Thus, the exact effects of plasma exchange therapy remain obscure. The most commonly used therapeutic agents other than corticosteroids are discussed as follows.

### 6.1. MTX

The ENMC guideline recommends the use of MTX with corticosteroids. In pediatric patients with IMNM, the dosage of MTX should be 0.3 mg/kg/week (maximum: 15 mg/week). However, a case series on pediatric anti-SRP myopathy reported that none of their patients (*n* = 3) responded to a combination therapy comprising a corticosteroid and MTX (48). Thus, aggressive combination therapy is required for anti-SRP myopathy.

### 6.2. IVIg

The initial dosage of IVIg for patients with IMNM is 2 g/kg/month for 3–6 months. IVIg is effective in treating IMNM, particularly anti-HMGCR myopathy. In some pediatric patients with anti-HMGCR myopathy, their symptoms improved and were successfully managed through IVIg monotherapy (6). A pediatric case series including patients with anti-HMGCR myopathy and those with anti-SRP myopathy reported that IVIg administration ensured a relative long disease remission period compared with the outcomes of a combination of pulse methylprednisolone therapy with MTX or etanercept (36). In addition to IVIg, subcutaneous immunoglobulins were found to be effective in adult patients with anti-HMGCR myopathy (74). Recently, the US Food and Drug Administration approved IVIg for dermatomyositis. Although the pathomechanism underlying IMNM may not be the exact same as that underlying dermatomyositis, IVIg appears to be effective in both diseases.

### 6.3. Rituximab

The recommended dosage of rituximab for IMNM is 750 mg/m^2^ (maximum: 1 g). In adult patients, rituximab was effective in only one-third of all patients with refractory anti-HMGCR myopathy and ineffective in the remaining two-thirds of the population (75). It is generally not used as the initial treatment option in pediatric patients with anti-HMGCR myopathy. Only one study reported the successful management of a pediatric patient, who responded poorly to MTX, using rituximab monotherapy (33). Conversely, in another study, rituximab exhibited prominent and sustained effects in most adult patients with anti-SRP myopathy (76). The benefits of rituximab have also been reported in some pediatric patients (45, 48).

### 6.4. Cyclophosphamide

Although the ENMC guideline does not recommend cyclophosphamide as a preferential treatment option for IMNM, its use is common in pediatric patients with IMNM. Cyclophosphamide is also used to treat severe juvenile dermatomyositis; the recommended dosage is 3 doses of 500 mg/m^2^ cyclophosphamide every 2 weeks and then 6 or 7 doses of 750 mg/m^2^ cyclophosphamide every 3 or 4 weeks (77). Unfortunately, its side effects, including cardiac and pulmonary toxicities, may aggravate cardiac and pulmonary problems in patients with anti-SRP myopathy; thus, the cardiac and pulmonary functions of patients receiving cyclophosphamide should be monitored closely. Regarding cyclophosphamide’s side effects associated with reproduction, a cumulative dosage of >7.5 g/m^2^ is a major risk factor for male infertility.^1^

## 7. Conclusion

Without timely diagnosis and aggressive treatment, irreversible muscle damage may be unavoidable in patients with IMNM. Most pediatric patients experience an insidious disease course with cutaneous manifestations and are often misdiagnosed with muscular dystrophy or dermatomyositis. Because of the similarity between IMNM and the aforementioned conditions in terms of clinical features, autoantibody analysis and muscle MRI are essential for accurate diagnosis. In addition, muscle pathology should be investigated for differential diagnosis. Compared with adult patients with IMNM, most pediatric patients often exhibit treatment resistance. On the basis of expert consensus and real-world data, the early administration of a combination of corticosteroids with immunosuppressants or IVIg is recommended for improved clinical outcomes.

## Author contributions

W-CL initiated the need for this article, revised it critically for important intellectual content, and gave critical comments. C-HW wrote the manuscript and created the included table. All authors contributed to the article and approved the submitted version.

## Conflict of interest

The authors declare that the research was conducted in the absence of any commercial or financial relationships that could be construed as a potential conflict of interest.

## Publisher’s note

All claims expressed in this article are solely those of the authors and do not necessarily represent those of their affiliated organizations, or those of the publisher, the editors and the reviewers. Any product that may be evaluated in this article, or claim that may be made by its manufacturer, is not guaranteed or endorsed by the publisher.

## Footnotes

^1^ https://www.j-sfp.org/fertility/man.html
